# Evaluation of Seasonal Variation on the Health Risks Using the Quantitative Microbial Risk Assessment Approach in a Wastewater Treatment Plant in Hamadan, Iran

**DOI:** 10.34172/jrhs.2023.111

**Published:** 2023-03-25

**Authors:** Melika Hooshmandi, Ashraf Mazaheri Tehrani, Majid Habibi Mohraz, Mostafa Leili, Mohammad javad Assari

**Affiliations:** ^1^Department of Occupational Health Engineering, School of Public Health, Hamadan University of Medical Sciences, Hamadan, Iran; ^2^Department of Environmental Health Engineering, School of Health, Kashan University of Medical Sciences, Kashan, Iran; ^3^Center of Excellence for Occupational Health Engineering, Occupational Health and Safety Research Center, Hamadan University of Medical Sciences, Hamadan, Iran; ^4^Research Center for Health Sciences, School of Public Health, Hamadan University of Medical Sciences, Hamadan, Iran

**Keywords:** Wastewater treatment plants, Quantitative microbial risk assessment, Staphylococcus aureus, Season

## Abstract

**Background:** Wastewater treatment plants (WWTPs) are a source of airborne bacterial contamination that can pose health risks to staff. The aim of this study was to evaluate seasonal variations in the health risks of exposure to *Staphylococcus aureus* bioaerosols using the quantitative microbial risk assessment (QMRA) approach in a WWTP in Hamadan, Iran.

**Study Design:** This was a descriptive cross-sectional study.

**Methods:** This study determined the emission concentrations of *S. aureus* bioaerosols in summer and winter. Then, the health risks of three exposure scenarios (the worker, field engineer, and laboratory technician) were evaluated using the QMRA approach. The bioaerosol samples were collected every 12 days in both summer and winter of 2021 with a nutrient agar using a single-stage cascade impactor (Quick Take 30, SKC Inc.) in both outdoor and indoor environments.

**Results:** The results demonstrated that in both seasons, *S. aureus* bioaerosol concentrations in outdoor and indoor environments were below the standard established by the American Conference of Governmental Industrial Hygienists (500 CFU/m^3^ ). While in summer, the annual infection risks and the disease burden for the three exposure scenarios in both outdoor and indoor environments were higher than the United States Environmental Protection Agency (≤10^-4^ pppy) and the World Health Organization (WHO) (≤10^-6^ DALYs pppy^-1^) benchmarks, respectively.

**Conclusion:** The findings provided high health risks for staff in the three exposure scenarios of an indoor environment, which should not be ignored, as well as emphasizing the use of the QMRA approach to estimate health risks caused by occupational exposure to bioaerosols and taking executive measures to protect staff working in the WWTPs.

## Background

 Bioaerosol is a group of airborne particles of viable or dead biological origin, including bacteria, fungi, viruses, pollen, and various antigens.^1^ The dispersion of pathogenic bioaerosols to the atmosphere through various sources such as humans, plants, and animals has a potential impact on the environment and human health. Bioaerosols can cause allergic and infectious diseases and sick building syndrome.^2^ Although the negative effects of bioaerosols on the environment are well known, our understanding of their epidemiological effects is limited.^3^ Workers can have respiratory exposures by inhalation^4^ and penetrating through the mucous membrane of the nose, ear, and eyes,^5^ as well as dermal exposures to the surface of solid waste and sludge that contains bioaerosols. In dermal exposures, these bioaerosols can be ingested by eating, drinking, and even smoking with those contaminated hands in the workplace.^6^ The wastewater treatment workers have globally shown a wide range of diseases such as weakness, allergies, asthma, infection, fever, gastrointestinal and respiratory or pulmonary problems, cancer, and some illnesses named sewage worker’s syndrome, thus working in a wastewater treatment plant (WWTP) can cause occupational diseases.^7,8^

 Potential challenges associated with bioaerosols in both outdoor and indoor environments include an incomplete understanding of the concentration levels of bioaerosols, individual markers of the species causing damage to health, and globally adoptable technologies for conducting sampling and analytical approaches. The other challenges are lack of appropriate enactments, absence of real-time monitoring of the effect of bioaerosol concentrations on most nations worldwide, epidemiological issues caused by bioaerosols, vital aspects of the bioaerosol cycle starting from emissions to environmental and health hazards, and challenges to overcome the limitations of bioaerosol research.^3^

 Depending on which processes are used for WWTPs to treat raw wastewater, a high amount of bacteria will be released into the surrounding air.^9^ The detection of a common presence and high concentration of *Staphylococcus aureus* bioaerosol in different WWTP environments indicates that high health risks of *S. aureus* bioaerosol in WWTPs can increase the infection risks of wastewater workers. *S. aureus* is one of the most common causes of bacteremia and infective endocarditis, which is significantly related to sewage worker’s syndrome. Therefore, the study of *S. aureus* bioaerosol should be given sufficient attention in WWTPs.^1^

 There is no dose-response relationship for every variation of bacteria. Due to the high variation of bioaerosols, occupational risk assessment is still difficult in such environments.^10^ Quantitative microbial risk assessment (QMRA) is a valuable and mechanistic approach to understanding and estimating the human health risk associated with exposure to particular microbial pathogens emitted from WWTPs.^11^ Considering that there is no threshold for occupational exposure through the inhalation of bioaerosols, assessing the health risks of exposure using QMRA is important to protect staff working in WWTPs. Thus, the current study sought to measure the concentration levels of bioaerosols and quantify the annual infection risk and disease burden associated with both outdoor and indoor environments by the QMRA approach in a WWTP in Hamadan Iran.

## Methods

###  WWTP description

**Figure 1:**
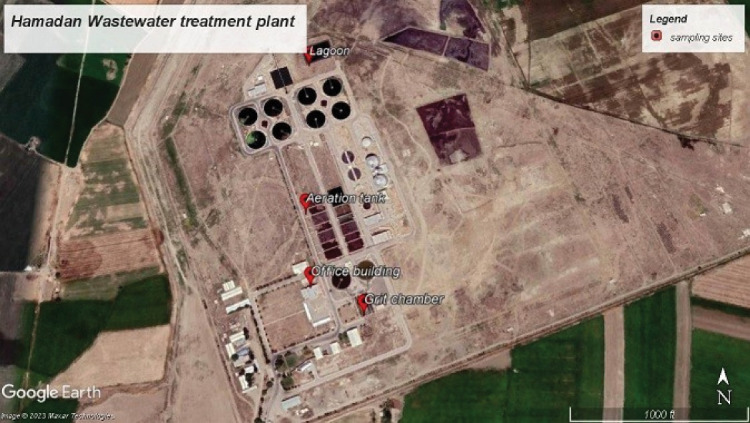
Sampling locations in Hamadan wastewater treatment plant

 Our experimental study took place in a WWTP in the northern part of Hamadan, one of the cities in the west of Iran. This WWTP has been working since 2011, and its inlet of swage was reported to be around 1.1 × 10^5^ m^3^/day for 500 000 inhabitants. The wastewater obtained after some chemical processes such as chlorination is used in irrigation systems, agriculture, and power plants. The sludge is also stored in the storage yard before being transported to be employed as compost in agriculture. The sampling sites are shown in Figure 1.

###  Sampling and analysis of bioaerosols

 In this work, three sites were selected, including an aerated grit chamber, aeration tank, and lagoon as the outdoor environment and inside of the office building as the indoor environment of the WWTP. Following the Environmental Protection Agency (EPA) sampling guidelines,^12^ air samples were collected every 12 days in both summer and winter in 2021. Finally, the number of samples in outdoor and indoor environments was 41 and 14, respectively. A single-stage cascade impactor (Quick Take 30, SKC Inc.), which has 400 drilled holes, was used to collect bioaerosol samples. The air samples were collected for 15 minutes with a flow rate of 28.3 L/min at 1.8 m above the ground surface and 1 m distant from the sites. The impactor was sterilized with 70% ethanol before and after each bioaerosol sampling to prevent contamination.^2^

 Bioaerosols were collected on 8 cm plates with a Nutrient Agar (NA) base as a non-selective medium for different viable airborne bacteria after autoclaving at 120^ᵒ^C for 15 minutes. After bioaerosol sampling, the plates were transferred to the laboratory using a cold box and incubated at 37^ᵒ^C for 48 hours.^2^ Then, the colonies were counted by using a Sana SL-902 colony counter, which was followed by Eq. (1)^13^ as:


Eq.(1) $$C=\frac{N\times 1000}{F\times t}$$

where *C* and *N* denote airborne bacterial concentrations in CFU/m^3^ and the total number of bacterial colonies in CFU, respectively. Moreover, *t* and *F* represent bioaerosol sampling time in minutes and the flow rate in L/min, respectively.

 The data were analyzed using SPSS (version 20) and expressed as means and standard deviations for numeric variables. An independent t-test was used to compare the total bacterial and the *S. aureus *concentration collected from outdoors and indoors during summer and winter. The *P *values of *≤ 0.05* were considered statistically significant.

###  Quantitative microbial risk assessment

 The QMRA approach was applied to evaluate and quantify the health risks (annual infection risk and disease burden) associated with exposure to microbial bioaerosols.^14^ The QMRA approach included four steps such as hazard identification, exposure assessment, dose-response assessment, and risk characterization,^15^ which are briefly described below:

####  Hazard identification

 The workers employed in the WWTP are subject to the risk of *S. aureus* inhalation because this is a well-known bioaerosol indicator, causing a large proportion of wastewater-associated illnesses.^1^ After identification, *S. aureus* was chosen as a bioaerosol indicator.

####  Exposure assessment

 In the present study, exposure assessment was used to estimate the dose of *S. aureus* bioaerosol that the staff employed in WWTPs were probably exposed within a day and a year. Therefore, a simplified and suitable approach, without considering the age of subjects as an influential factor, was utilized to estimate the exposure assessment.^16^ The exposure staff were divided into three scenarios, including workers, field engineers, and laboratory technicians (Table 1). Many factors such as temperature, wind speed, relative humidity, and solar radiation can influence the exposure level of staff to *S. aureus* bioaerosol, thus they were recorded simultaneously. The exposure dose of the microbial bioaerosol was estimated using Eq. (2):


Eq.(2) d=C×ag×br×t

where *d* is the exposure dose expressed in pathogens day^-1^, and *C* denotes the concentration of the calculated bacterial bioaerosol (CFU/m^3^) using Eq. (1). In addition, ag represents the aerosol ingestion rate (%) that was considered 0.1-0.5,^17^ and br is the breathing rate (m^3^/day) that its range is 0.588-0.780 m^3^/d and 0.575-0.604 m^3^/d in men and females, respectively.^18^ Further, *t *is the exposure time (h/day) that was separately estimated for each of the three investigated scenarios (Table 1).

**Table 1:** Description of staff’s categories exposed to bioaerosols in Hamadan wastewater treatment plant

**Staffs Categories**	**Worker**	**Field Engineer**	**Laboratory Technician**
Male staffs	9	8	0
Female staff’s	0	0	2
Exposure condition outdoor (hour/day)	9	3	1
Exposure condition indoor (hour/day)	3	5	7
Exposure time (day/week)	3	6	6
Mean exposure frequency (day/year)	240	270	270

####  Dose-response assessment

 The exponential dose-response model as a dose-infection model for the *S. aureus *bioaerosol was used to determine the relationship between the dose and the infection risks.^14,19,20^ This model is expressed by Eq. (3):


Eq.(3) $$P_{inf}=1\;-\;e^{-rd}$$

where *P*_inf_ and d represent the probability of being infected after daily exposure to pathogens (per person per day) and the exposure dose calculated in Eq. (2) (pathogens day^-1^), respectively. Furthermore, *r* is the parameter related to the infectivity constant of the *S. aureus* bioaerosol in the exponential dose-response or dose-infection model (unit-less), which was considered 6.46E08-1E-07 according to the value reported in related studies.^21^ Finally, ddenotes the exposure dose computed in Eq. (2) (pathogens day^-1^).

 The annual infection risks were calculated based on the theorem of independence^22^ using Eq. (4):


Eq.(4) $$P_{a\left(inf\right)}=1-{\left(1-P_{inf}\right)}^{n},$$

where *P*_a(inf)_ and *P*_inf_ indicate the probability of being infected after a yearly exposure expressed in per person per year (pppy) and after daily exposure (per person per day), respectively. In addition, n is the exposure frequency per person in days per year (day^-1^) that was separately determined for each of the three investigated scenarios (Table 1).

####  Risk characterization

 It was performed based on the health risks, including annual infection risk and disease burden for staff.^11^ The specific potential disease burden (DALYs pppy) caused by exposure to the *S. aureus* bioaerosol was estimated in Eq. (5):


Eq.(5) $$DB=P{\;}_{a\left(inf\right)}\times P_{ill/inf}\times HB,$$

where DB is the disease burden expressed in DALYs pppy (DALYs pppy^-1^). Further, *P*_a(inf)_, *P*_ill/inf_, and HB denote the annual infection risk (pppy), the probability of the illness-to-infection ratio, and the disease burden per case (DALYs per case), respectively. The results of health risks were characterized according to the United States (U.S.) EPA annual probability of infection (≤ 10^-4^ pppy) and the World Health Organization (WHO) disease burden (≤ 10^-6^ DALYs pppy^-1^) benchmarks.^11,23,24^

####  Model implementation

 The Monte-Carlo simulation technique was used to represent the propagation of variability in QMRA.^17^ It was run for 10000 iterations for each distribution of inputted variables such as exposure concentration, three input exposure parameters (exposure time, aerosol ingestion rate, and breathing rate), and the dose-response/dose-infection model) of the *S. aureus* bioaerosol.

## Results

 The total bacterial and *S. aureus* counts recovered in bioaerosol samples collected from outdoor and indoor environments of the WWTP during summer and winter are presented in Table 2. Based on the results, in winter, the mean total bacterial and *S. aureus* bioaerosol concentrations in both outdoor and indoor environments, to a considerable extent, were below the standard established by American Conference of Governmental Industrial Hygienists (ACGIH, 500 CFU/m^3^), while in summer, the mean total bioaerosol concentration in the outdoor environment was beyond the mentioned standard.

**Table 2:** Total bacterial and *Staphylococcus aureus* concentration counts recovered (CFU/m^3^) in summer and winter seasons

	**Total Bacterial**					**Staphylococcus aureus**				
	**Winter**		**Summer**		***P *** **value**	**Winter**		**Summer**		***P *** **value**
**Environment**	**Mean**	**SD**	**Mean**	**SD**		**Mean**	**SD**	**Mean**	**SD**	
Outdoor	65.63	68.79	623.03	488.32	0. 001	4.51	8.06	127.19	221.33	0.010
Indoor	61.64	57.35	470.70	316.76	0.100	4.12	7.75	111.90	150.72	0.010

*Note*. SD: Standard deviation.

 The results of the QMRA approach (Table 3) based on the calculation of annual infection risks and the disease burden of the *S. aureus* bioaerosol for the three exposure scenarios (the worker, field engineer, and laboratory technician) revealed that for the indoor environment, staff’s health risks in summer were about 100 times greater than in winter. Moreover, the health risks estimated for the indoor environment in both summer and winter for laboratory technicians were about 1.3 and 2 times greater than those estimated for field engineers and workers, respectively (Table 3).

**Table 3:** Calculated of quantitative microbial risk assessment based on annual infection risks (× 10^-4^ pppy) and disease burden (× 10^-6^ DALYs pppy)

**Categories**	**Health Risks**	**Workers**	**Field Engineer**	**Laboratory Technician**
Summer				
Outdoor	P_y_	6.309	2.398	1.000
	DB	1.621	0.631	0.257
Indoor	P_y_	11.749	18.198	24.547
	DB	3.090	4.677	6.310
Winter				
Outdoor	P_y_	1.175	0.456	0.186
	DB	0.302	0.114	0.048
Indoor	P_y_	0.107	0.162	0.218
	DB	0.027	0.043	0.056

*Note*. P_y_: Annual infection risks; DB: Disease burden.

 The results further indicated that in summer, the annual infection risks for the three exposure scenarios in both outdoor and indoor environments were higher than the U.S. EPA annual infection benchmark (≤ 10^-4^pppy), while in winter, except for the worker group in outdoor environments, the infection risks of other scenarios in both outdoor and indoor environments were below the mentioned benchmark. Meanwhile, in summer, the disease burden for the worker group only in the outdoor environment and in all exposure scenarios in the indoor environment exceeded the WHO disease burden benchmark (≤ 10^-6^DALYs pppy^-1^). Moreover, the findings showed that in summer, the health risks estimated in the indoor environment for the laboratory technician group were clearly beyond the U.S. EPA and the WHO benchmarks for the annual infection risks and the disease burdens, respectively (Table 3).

## Discussion

 Based on data (Table 2), the mean total bacterial and *S. aureus* bioaerosol concentrations in both outdoor and indoor environments were below 500 CFU/m^3^ in winter, which can be classified as an uncontaminated level according to the ACGIH standard.^25^ This finding might be due to the higher average temperature in summer, which could significantly affect the concentration of bioaerosols in WWTPs.^26^ However, no internationally accepted threshold level exists because bioaerosols are complex mixtures of microbial particles,^27,28^ and local levels are different from 800 CFU/m^3^to 1 × 10^4^ CFU/m^3^ as established by Korea and Switzerland, respectively.^29-31^ Additionally, it is worth noting that these Occupational Exposure Limits are usually recommended on simple baseline bioaerosol concentrations rather than dose-response relationships of health risk assessment, thus the threshold values for bioaerosol concentrations are still not practical because of the limited data and intrinsic variability to each individual.^28^

 The results of the present study indicated that in summer, the annual infection risks and the disease burden for the three exposure scenarios in both outdoor and indoor environments were higher than the U.S. EPA and WHO benchmarks, respectively. The findings in Table 2 were caused by the significant difference (*P <*0.05) between the average *S. aureus *concentration in both seasons. Previous studies reported that the annual infection risks are commonly expressed based on the dose of exposure to microbial bioaerosol concentrations,^32^ Similar results also demonstrated a significant association between exposure to microbial bioaerosol concentrations and health risk by other researchers.^33-35^

 The source of indoor bioaerosols can be from outdoor air, furniture, plants, organic wastes, and human activities such as speaking, strolling, coughing, sneezing, and the like.^36^ Furthermore, air exchange with the outdoor environment and building conditions can affect bioaerosols’ existence.^36^ Even indoor human-associated bioaerosols such as staphylococcus can be more than outdoor bioaerosols.^36^ Although in this study, the mean of *S. aureus* concentrations in the indoor environment was less than in the outdoor environment (Table 2), in the indoor environment, the staff’s health risks in summer were significantly greater than in winter (Table 3). This finding is related to differences in health risks, indicating the positive effect of temperature on bioaerosol’s growth and its transfer into the environment.^2^

 The findings (Table 3) further revealed that in summer, the sequence of health risks estimated in the indoor environment for the three groups of staff was the laboratory technician > field engineer > worker. Referring to the three exposure scenarios, the health risks of the laboratory technician group were the highest. The next susceptible group consisted of the field engineers, and the workers group that was at the lowest risk (Table 3). Thus, the laboratory technicians were more vulnerable and their health risks would be spontaneously higher than those of the other groups. In the present study, two females were working in the WWTP as laboratory technicians. Several studies reported that the average breathing rate of males is generally higher than that of females, and the health risks are then associated with this elevated exposure dose, thus, as expected, the health risks of males are always higher than those of females.^32,37,38^ This contradiction could be attributed to the exposure time in the indoor environment for laboratory technicians in the WWTP (7 hours/day) that was longer than for two other exposure scenarios (Table 1). This finding is in agreement with the findings of similar studies, representing a significant positive relationship between the time of exposure and the health risks^39^ and verifying a trend that the health risks increase with the exposure time.^40^

 In the present study, due to the limitation in the number of staff, it was impossible to compare the health risk in different scenarios through statistics tests. Moreover, when calculating health risks, the related characteristics of different age groups of participants were not taken into account. Therefore, this evaluation might not best characterize the true impact of health risks related to bacterial bioaerosols. In fact, disease surveillance databases, which are based on surveillance data from various regions of the world, are needed for a more accurate and reliable health risk assessment.^32^

**Highlights**

- The mean of S. aureus bioaerosol concentrations in summer was higher than in winter.
- The health risks of three exposure scenarios in summer were higher than the benchmark.
- Staff’s health risks outdoors and indoors were greater in summer than in winter.

## Conclusion

 The findings showed that in summer, the annual infection risks and the disease burden for the three exposure scenarios of the indoor environment were to a considerable extent higher than in the outdoor environment. Therefore, these results represented high health risks for the staff of the WWTP, which could not be ignored. As a result, considering the poor hygienic measures applied by staff in the WWTP, equipping workers with appropriate masks such as N99 and effective management prevention measures must be implemented to minimize the generation of microbial bioaerosols’ exposure dose in the workplace to reduce health risks. Finally, the use of the QMRA approach in studying airborne microbes can estimate the health risks caused by exposure to bioaerosols and offer proposals that could be executed by authorities to protect staff working in WWTP health.

## Acknowledgements

 This study was part of an M.Sc. thesis in occupational health engineering and was conducted with the approval of Hamadan University of Medical Sciences, Medical Ethics Committee (Code: IR.UMSHA.REC.1399.713). Thanks are owed to the Vice-chancellor for Research and Technology of Hamadan University of Medical Sciences for their help in conducting this study.

## Competing Interests

 The authors declare that they have no competing interests.

## Funding

 The present study was supported by the Hamadan Province Water and Wastewater Company under Grant [99/137].
